# Analysis of recently identified dyslipidemia alleles reveals two loci that contribute to risk for carotid artery disease

**DOI:** 10.1186/1476-511X-8-52

**Published:** 2009-12-01

**Authors:** James Ronald, Ramakrishnan Rajagopalan, Jane E Ranchalis, Julieann K Marshall, Thomas S Hatsukami, Patrick J Heagerty, Gail P Jarvik

**Affiliations:** 1 Department of Medicine, Division of Medical Genetics, University of Washington, Seattle, WA, USA; 2 Department of Surgery, Division of Vascular Surgery, University of Washington, Seattle, WA, USA; 3 Department of Biostatistics, University of Washington, Seattle, WA, USA

## Abstract

**Background:**

Genome-wide association studies have identified numerous single nucleotide polymorphisms (SNPs) affecting high density lipoprotein (HDL) or low density lipoprotein (LDL) cholesterol levels; these SNPs may contribute to the genetic basis of vascular diseases.

**Results:**

We assessed the impact of 34 SNPs at 23 loci on dyslipidemia, key lipid sub-phenotypes, and severe carotid artery disease (CAAD) in a case-control cohort. The effects of these SNPs on HDL and LDL were consistent with those previously reported, and we provide unbiased estimates of the percent variance in HDL (3.9%) and LDL (3.3%) explained by genetic risk scores. We assessed the effects of these SNPs on HDL subfractions, apolipoprotein A-1, LDL buoyancy, apolipoprotein B, and lipoprotein (a) and found that rs646776 predicts apolipoprotein B level while rs2075650 predicts LDL buoyancy. Finally, we tested the role of these SNPs in conferring risk for ultrasonographically documented CAAD stenosis status. We found that two loci, chromosome 1p13.3 near CELSR2 and PSRC1 which contains rs646776, and 19q13.2 near TOMM40 and APOE which contains rs2075650, harbor risk alleles for CAAD.

**Conclusion:**

Our analysis of 34 SNPs contributing to dyslipidemia at 23 loci suggests that genetic variation in the 1p13.3 region may increase risk of CAAD by increasing LDL particle number, whereas variation in the 19q13.2 region may increase CAAD risk by promoting formation of smaller, denser LDL particles.

## Background

Carotid artery disease (CAAD) is an important risk factor for stroke, the third leading cause of death in the U.S. Given the high mortality, morbidity, and economic costs due to stroke, primary prevention, particularly targeted toward high risk individuals, is the most promising approach to combat stroke[1,2]. Although medical interventions and carotid endarterectomy can potentially prevent strokes in individuals with CAAD, routine screening is not currently recommended[1]. However, it has been suggested that if high risk groups with CAAD prevalences of approximately 20% can be identified, screening may provide significant and cost effective extension to quality adjusted life years[3,4]. Studies of siblings[5,6], twins[7,8], and families[9] suggest a heritable genetic contribution to carotid artery intima-media thickening and stenosis from plaque, with the heritability of ultrasonographically measured phenotypes typically ranging from 20% to 40% in population based samples[10]. Thus, identification of genetic risk factors for carotid artery stenosis, progression, and plaque instability may ultimately be useful in targeting primary prevention against stroke in patients for whom management strategies are not yet well defined.

Recently a number of large genome-wide association studies have revealed loci affecting total cholesterol, high-density lipoprotein cholesterol (HDL), low-density lipoprotein cholesterol (LDL), and triglycerides [11-15]. Because of their role in promoting dyslipidemia, these single nucleotide polymorphisms (SNPs) are strong candidates for contributing to genetic risk for atherosclerosis, and several studies have found significant impacts of these loci on coronary artery disease[11,12,16]. Although many clinical risk factors such as age, smoking, hypertension, and diabetes are shared between CAAD and coronary artery disease, the relative importance of these risk factors differs between these two vascular disease processes [17]. Similarly, the relative importance of risk factors varies for disease at different locations within the carotid arteries themselves[9,10]. These discrepancies suggest that additional factors, including genetic ones, may modulate the atherosclerotic disease process differently in different anatomic locations. Thus, the impact of recently discovered dyslipidemia risk alleles on CAAD is as yet unknown.

Based on previous success in applying genetic risk scores for decreased HDL and increased LDL to the prediction of coronary artery disease[12] and the central role of these lipid fractions in evidence-based guidelines for coronary artery disease risk reduction[18], we investigated the role of SNPs affecting HDL and LDL in predicting risk for CAAD. We also sought to determine whether these SNPs alter key lipid sub-phenotypes with differential atherogenic potential. Specifically, the more efficient cholesterol efflux activity of apolipoprotein A-I (apo A-I) [19] has lead to the hypothesis that the HDL_2 _subfraction or apo A-I may be a better predictor of protection against atherosclerosis than HDL_3 _or total HDL. We also tested SNPs for their effects on apolipoprotein B (apo B), which measures LDL particle number and may be a better estimator of cardiovascular disease risk than LDL level[20,21], LDL buoyancy which predicts the smaller, denser LDL pattern B phenotype [22] associated with increased risk of coronary artery disease[23], and lipoprotein(a) (Lp(a)) which appears to independently predict risk of coronary artery disease [24] and stroke [25]. These analyses may suggest mechanisms through which specific SNPs modulate CAAD risk beyond their effects on HDL and LDL levels.

## Results

### Effects of SNPs on HDL and LDL

**Figure 1:**
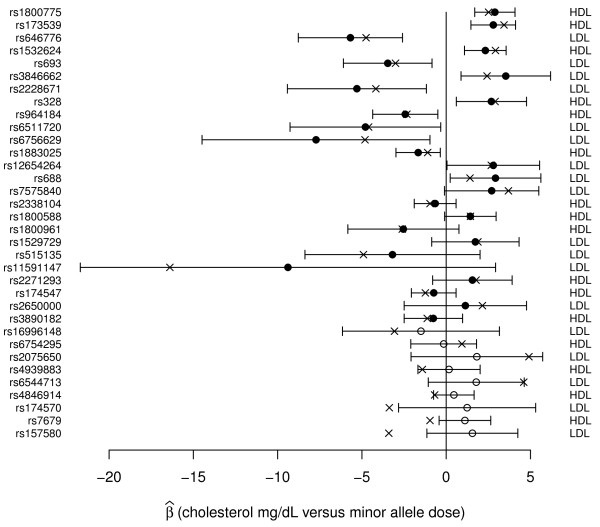
**Effects of SNPs on HDL and LDL**. The estimated regression coefficient (circles) for the effect of each SNP on HDL or LDL (listed on the right) is shown with the associated 95% confidence interval. Previously reported effects from the literature are marked with x's. SNPs are sorted by decreasing relative likelihood under the alternative ( as given by the x) versus the null hypothesis ( = 0) with closed circles indicating SNPs for which the data are more likely under the alternative hypothesis.

As shown in Figure 1 our data confirm the previously reported effects on HDL and LDL for the majority of SNPs tested in CLEAR study participants (see Table 1). Out of 34 SNPs tested (see Table 2), we identified 14 SNPs that showed nominally significant associations with HDL or LDL levels at a p-value of 0.05, corresponding to a FDR of 0.11 when corrected for multiple testing. These 14 SNPs correspond to those for which the 95% confidence intervals do not cross zero in Figure 1. For only three SNPs the 95% confidence intervals do not contain the previously reported effect from the literature, and for the top 25 SNPs, those indicated by closed circles, our data are more likely assuming the effect as reported in the literature (i.e. 's as given by the x's in Figure 1) than under the null ( = 0). Furthermore, for 28 out of 34 SNPs the effects on HDL or LDL in our data were in the same direction as reported previously in the literature, corresponding to significant concordance under a binomial test (p = 2.0 × 10^-4^).

**Table 1:** Characteristics of CLEAR participants.

	**Controls ≤15% stenosis** **(N, 25^**th**^/50^**th**^/75^**th **^percentiles, or %)**	**50-79% stenosis**	**Cases ≥80% stenosis**	**P-value**^**a**^
N	479	83	353	
Censored age	60/66/72	66/71/76	59/66/72	0.30
Current smoker	9.5%	18%	33%	2.1 × 10^-16^
Pack-years smoked	0.0/5.2/30	8.8/32/68	15/45/69	6.1 × 10^-36^
Body mass index (BMI)	25/28/31	24/27/30	24/27/30	0.0025
Anti-hypertensive medication use	49%	86%	84%	5.9 × 10^-25^
Diabetes	18%	29%	34%	4.8 × 10^-8^
Lipid lowering medication use	22%	69%	68%	7.9 × 10^-40^
HDL (mg/dL)	39/47/57	35/40/50	35/41/49	5.2 × 10^-11^
LDL (mg/dL)	97/115/134	86/102/126	86/102/125	1.3 × 10^-5^

^a^P-values are derived from t-tests for the difference in means or χ^2 ^tests for the difference in percentages between controls (≤15% stenosis) and cases (≥80% stenosis).

**Table 2:** SNPs analyzed in the CLEAR study.

**SNP**	**Chr**	**Base Pair**	**Nearby Gene(s)**	**Minor (Major) Allele**	**Change in HDL/LDL per copy of minor allele (mg/dL)**	**Genotyped/Imputation Accuracy**^**f**^
rs11206510^ab^	1	55268627	PCSK9	C (T)	-2.8 LDL	0.55
rs11591147^cd^	1	55278235	PCSK9	T (G)	-16.4 LDL	Genotyped
rs12740374^a^	1	109619113	CELSR2-PSRC1-SORT1	T (G)	-7.1 LDL	0.97^g^
rs646776^de^	1	109620053	CELSR2-PSRC1-SORT1	G (A)	-4.8 LDL	Genotyped
rs4846914^ad^	1	228362314	GALNT2	G (A)	-0.7 HDL	Genotyped
rs6754295^e^	2	21059688	APOB	C (A)	0.9 HDL	Genotyped
rs693^bcde^	2	21085700	APOB	G (A)	-3.0 LDL	Genotyped
rs7575840^c^	2	21126995	APOB	T (G)	3.7 LDL	Genotyped
rs515135^a^	2	21139562	APOB	T (C)	-4.9 LDL	0.90
rs6756629^e^	2	43918594	ABCG5	A (G)	-4.8 LDL	Genotyped
rs6544713^a^	2	43927385	ABCG8	T (C)	4.6 LDL	1.00
rs12654264^cd^	5	74684359	HMGCR	T (A)	2.7 LDL	Genotyped
rs3846662^e^	5	74686840	HMGCR	G (A)	2.4 LDL	Genotyped
rs3846663^a^	5	74691482	HMGCR	T (C)	2.1 LDL	0.97^h^
rs1501908^a^	5	156330747	TIMD4-HAVCR1	G (C)	-2.1 LDL	0.11
rs12670798^c^	7	21573877	DNAH11	G (A)	2.7 LDL	0.18
rs328^cd^	8	19864004	LPL	G (C)	2.9 HDL	Genotyped
rs10096633^e^	8	19875201	LPL	A (G)	1.9 HDL	0.94^i^
rs12678919^a^	8	19888502	LPL	G (A)	3.2 HDL	0.94^i^
rs2083637^e^	8	19909455	LPL	G (A)	1.5 HDL	0.45
rs471364^a^	9	15279578	TCC39B	C (T)	-1.1 HDL	No genotyped SNPs within 200 kb
rs3890182^cd^	9	106687476	ABCA1	A (G)	-1.1 HDL	Genotyped
rs3905000^e^	9	106696891	ABCA1	A (G)	-1.6 HDL	0.98^j^
rs1883025^a^	9	106704122	ABCA1	T (C)	-1.1 HDL	Genotyped
rs7395662^e^	11	48475469	MADD-FOLH1	A (G)	1.0 HDL	No genotyped SNPs within 200 kb
rs174547^a^	11	61327359	FADS1-FADS2-FADS3	C (T)	-1.2 HDL	1.00
rs174570^e^	11	61353788	FADS1-FADS2-FADS3	A (G)	-3.4 LDL	Genotyped
rs964184^a^	11	116154127	APOA1-APOC3-APOA4-APOA5	G (C)	-2.3 HDL	1.00
rs2338104^ab^	12	108379551	MMAB-MVK	C (G)	-1.0 HDL	Genotyped
rs2650000^a^	12	119873345	HNF1A	A (C)	2.1 LDL	0.98
rs10468017^a^	15	56465804	LIPC	T (C)	1.4 HDL	0.029
rs1532085^e^	15	56470658	LIPC	A (G)	1.8 HDL	0.17
rs1800588^cd^	15	56510967	LIPC	T (C)	1.4 HDL	Genotyped
rs173539^a^	16	55545545	CETP	T (C)	3.4 HDL	1.00
rs1800775^cd^	16	55552737	CETP	A (C)	2.5 HDL	Genotyped
rs1532624^e^	16	55562980	CETP	A (C)	2.9 HDL	Genotyped
rs2271293^ae^	16	66459571	LCAT-CTCF-PRMT8	A (G)	1.8 HDL	0.98
rs4939883^ae^	18	45421212	LIPG	A (G)	-1.4 HDL	0.92
rs2967605^a^	19	8375738	ANGPTL4	T (C)	-1.6 HDL	0.76
rs1529729^c^	19	11024562	LDLR	C (T)	1.9 LDL	Genotyped
rs6511720^abc^	19	11063306	LDLR	T (G)	-4.6 LDL	Genotyped
rs2228671^e^	19	11071912	LDLR	A (G)	-4.2 LDL	Genotyped
rs688^c^	19	11227602	LDLR	T (C)	1.4 LDL	Genotyped
rs10401969^a^	19	19268718	NCAN-CILP2-PBX4	C (T)	-1.5 LDL	0.22
rs16996148^bd^	19	19519472	NCAN-CILP2-PBX4	T (G)	-3.1 LDL	Genotyped
rs157580^e^	19	50087106	TOMM40-APOE	G (A)	-3.4 LDL	Genotyped
rs2075650^e^	19	50087459	TOMM40-APOE	G (A)	4.9 LDL	Genotyped
rs4420638^abc^	19	50114786	APOE	G (A)	8.9 LDL	0.81
rs6102059^a^	20	38662198	MAFB	T (C)	-1.8 LDL	-0.074
rs1800961^a^	20	42475778	HNF4A	T (C)	-2.6 HDL	Genotyped
rs7679^a^	20	44009909	PLTP	C (T)	-1.0 HDL	Genotyped

The estimated changes in HDL or LDL per copy of the minor allele are based on effects reported in ^a^Kathiresan et al[14], ^b^Willer CJ et al[15], ^c^Kathiresan et al[12], ^d^Kathiresan et al[13], and ^e^Aulchenko et al[11]. ^f^Only SNPs with estimated imputation accuracies of >90% were included in downstream analyses. ^g^Excluded from downstream analyses due to r^2 ^= 1.00 with rs646776. ^h^Excluded due to r^2 ^= 0.99 with rs12654264. ^i^Excluded due to r^2 ^= 0.93 and 0.95 with rs328. ^j^Excluded due to r^2 ^= 0.96 with rs3890182.

Given the small marginal effects of the recently reported SNPs on HDL or LDL levels, we used a genetic risk score combining alleles additively across SNPs to better predict lipid levels. Such genetic risk scores have previously been utilized, but they are subject to upward bias when developed in the same sample used to initially detect associations. Using a risk score with SNPs weighted by the reported effect sizes from the literature (see Table 2) and after accounting for covariates, the 16 HDL SNPs explain 3.9% of the variation in HDL levels (p = 4.3 × 10^-9^). Using the same approach, the 18 LDL SNPs explain 3.3% of the variation in LDL levels (p = 4.4 × 10^-8^).

### Effects of SNPs on lipid sub-phenotypes

With few exceptions we found that the same SNPs significantly associated with total HDL in our data were also associated with HDL_2_, HDL_3_, and apo A-I. At a p-value of 0.05, corresponding to an FDR of 0.11, we identified 19 significant associations between the 16 HDL SNPs and HDL_2_, HDL_3_, or apo A-I. In a principal component analysis of the t-statistics derived from tests of association between the 16 HDL SNPs and 4 phenotypes (total HDL, HDL_2_, HDL_3_, and apo A-I), we found that the first principal component was positively correlated with all 4 phenotypes and explained 94% of the variance in the t-statistics, indicating that the effects of each SNP were highly concordant across all HDL related phenotypes. Similarly, the hierarchical clustering analysis performed by Kathiresan et al[14] also shows that the current set of genetic loci affecting HDL does little to discriminate among HDL_2_, HDL_3_, and apo A-I levels.

At a p-value of 0.05, we found that rs646776, rs693, rs2228671, and rs6511720 were associated with apo B and that rs2075650 and rs2650000 were associated with LDL buoyancy. The associated FDR for these tests was quite high at 0.35, due in part to the absence of any affect of these SNPs on Lp(a). Among SNPs associated with apo B, rs646776 was by far the most statistically significant (p = 0.00035, compared with 0.017 ≤ p < 0.05 for the remaining three) with its minor allele decreasing apo B ( = -3.3). Kathiresan et al[14] found a similarly strong effect on apo B of the nearby SNP rs12740374 ( = -3.3; p = 1.2 × 10^-8^), which is nearly perfectly correlated with rs646776 (see Table 2). Among SNPs associated with LDL buoyancy, rs2075650 was the most statistically significant (p = 0.014), with the minor allele decreasing the relative flotation rate ( = -0.0047) leading to a more atherogenic phenotype.

### Effects of SNPs on CAAD risk

**Figure 2:**
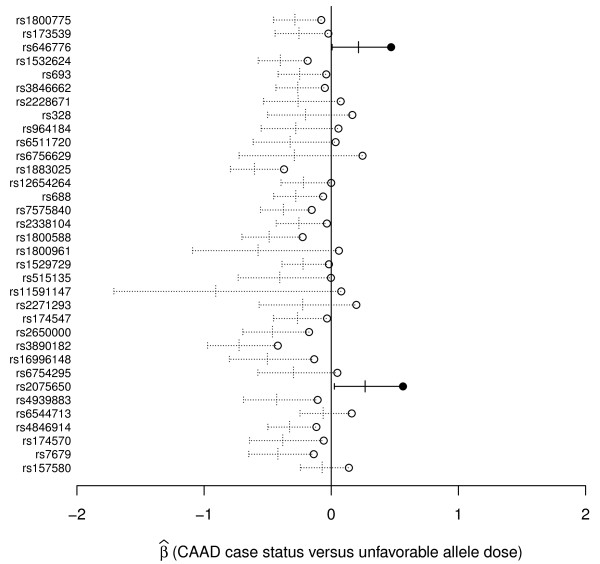
**Effects of SNPs on CAAD risk**. The estimated coefficient (circles) for each SNP in a logistic regression model of CAAD case versus control status is shown along with the associated 95% (longer vertical ticks) and 99.9% (shorter vertical ticks) confidence intervals, corresponding to one-sided p-values of 0.05 and 0.0015 respectively. Closed circles with confidence intervals in solid lines represent those SNPs that are significantly associated with CAAD after a Bonferroni correction for 34 tests.

After a Bonferroni correction for 34 tests, corresponding to a threshold of p = 0.0015, Figure 2 shows that two SNPs, rs646776 and rs2075650, met criteria for significant association with CAAD. For convenience SNPs have been recoded from minor allele dose to unfavorable allele dose based on their effect on HDL or LDL so that the expected effect on CAAD is  >0. Due to the prior expectation that alleles that decrease HDL or increase LDL would confer increased risk for CAAD, we performed one-sided tests. The major allele of rs646776 is associated with increased LDL, increased apo B as described above, and increased risk of CAAD ( = 0.47; p = 0.0012). The minor allele of rs2075650 is associated with increased LDL, decreased LDL buoyancy as described above, and increased risk of CAAD ( = 0.56; p = 0.00091). Results were similar when we analyzed percent carotid stenosis (with controls, individuals with intermediate stenosis, and cases having phenotypes coded 0, 0.5, and 0.8 respectively) in a linear regression allowing for inclusion of 83 additional individuals with intermediate carotid stenosis with luminal narrowing of 50 to 79% (p = 0.0049 for rs646776, p = 0.0010 for rs2075650).

The associations between rs646776 and rs2075650 and CAAD could not be explained by their effects on HDL and LDL levels alone. We found that including the observed HDL and LDL levels, use of lipid lowering therapy, or "pre-therapy" HDL and LDL levels (see Methods) as covariates did not dramatically change the significance or magnitude of the associations between CAAD and rs646776 or rs2075650 (data not shown). Combined with their effects on LDL particle number and buoyancy, these results raise the possibility that rs646776 and rs2075650 contribute additional information to current HDL and LDL levels alone for CAAD risk prediction.

### Genetic risk score for CAAD

Contrary to HDL and LDL, we did not find that CAAD risk prediction was improved using an additive risk score based on these SNPs. We tested both a risk score in which all SNPs were weighted equally as in Kathiresan et al[12] (p = 0.32), and one in which SNPs were weighted by the magnitude of their effects on HDL and LDL as in Aulchenko et al[11] (p = 0.63). Results were similar with or without HDL, LDL, and use of lipid lowering as covariates. To clarify this finding, we sought to determine our power to detect association between the genetic risk score and CAAD, given that the risk score based on these SNPs explains only 3.9% and 3.3% of the variance in HDL and LDL levels, respectively, and given that dyslipidemia is only one of several risk factors for CAAD. Using power simulations (see Methods), we found that the genetic risk score, acting through its effects on HDL and LDL levels alone, had an expected odds ratio for association with CAAD of 1.03 to 1.04 per unfavorable allele. The power based on these effect sizes was only 20% to 39%, suggesting that we cannot reject a model in which the overall genetic score confers risk of CAAD, but mainly through its effect on lipid levels. However, if the odds ratio per unfavorable allele averaged as little as 1.07 to 1.10 our power to detect association between the risk score and CAAD was 81% to 97%. This suggests that if a significant fraction of these 34 SNPs are predictive of CAAD risk beyond their effects on measured HDL and LDL levels, we would have had high power to detect such an association.

### CAAD risk locus on chromosome 1p13.3

**Figure 3:**
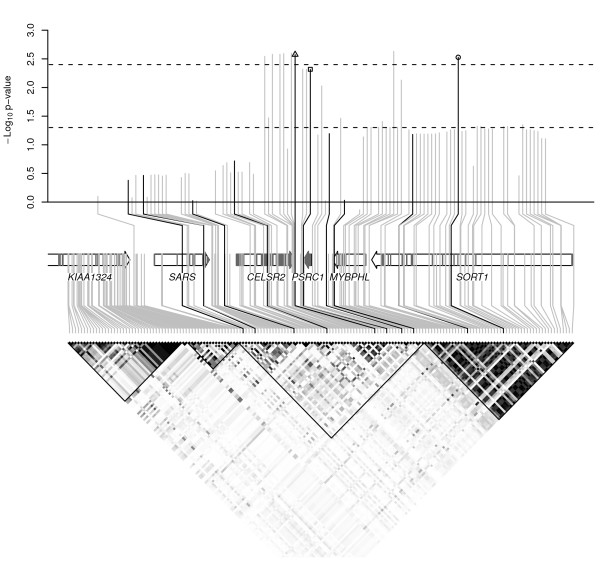
**Linkage disequilibrium structure and association results for the chromosome 1p13.3 region containing rs646776**. The lower portion of the figure shows pairwise linkage disequilibrium in HapMap individuals with white indicating r^2 ^= 0 and black indicating r^2 ^= 1. Approximate boundaries of linkage disequilibrium blocks are traced. The locations of genotyped and imputed SNPs relative to the genes in the region (middle portion) are shown with black and light gray lines, respectively. Exonic regions of genes are shaded dark gray. The upper portion shows -log_10 _p-values for association with CAAD, with nominal significance (p = 0.05) and permutation corrected significance levels for 83 analyzed SNPs indicated by the lower and upper dashed lines, respectively. rs646776, rs599839, and rs4970843 are marked by the open triangle, square, and circle, respectively.

To further explore the CAAD association in the vicinity of rs646776 we analyzed 82 additional SNPs that were genotyped or imputed with at least 80% accuracy, and Figure 3 shows that rs646776 and neighboring SNPs in CELSR2 and PSRC1 exhibit the greatest statistical significance. To correct for multiple testing in the setting of strong linkage disequilibrium we performed permutation testing to estimate a significance threshold of p = 0.0040. Within SORT1 the intronic SNP rs4970843 shows significant association (p = 0.0030). Interestingly rs4970843 is in weak but significant long range linkage disequilibrium with rs646776 (r^2 ^= 0.083; p = 2.5 × 10^-19^), and is in relatively weaker linkage disequilibrium with nearby SNPs in the highly correlated block encompassing SORT1.

### CAAD risk locus on chromosome 19p13.2

**Figure 4:**
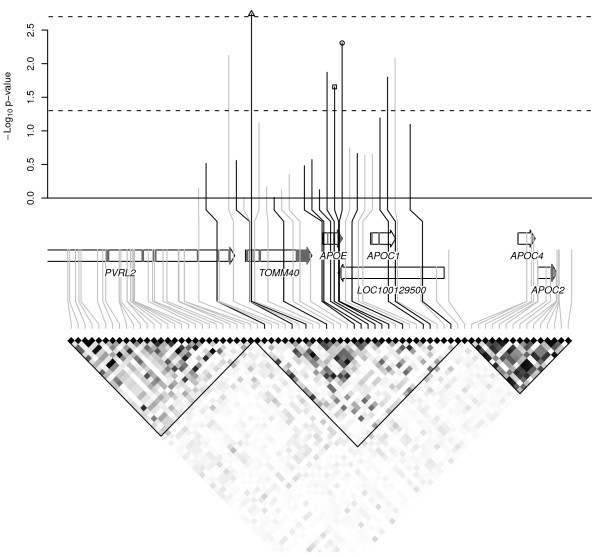
**Linkage disequilibrium structure and association results for the chromosome 19p13.2 region containing rs2075650**. See the Figure 3 caption for further description. The upper dashed line represents a Bonferroni correction for 25 analyzed SNPs. rs2075650, rs429358, and rs7412 are indicated by the open triangle, square, and circle, respectively.

Figure 4 shows that rs2075650, an intronic SNP in TOMM40, displays stronger association with CAAD than rs429358, which defines the ε3/ε4 dichotomy, or rs7412, which defines the ε2/ε3 dichotomy, in the APOE ε2/ε3/ε4 polymorphism. When rs2075650 and rs429358 were analyzed conditional on one another, we found significant association with rs2075650 (p = 0.032) but not with rs429358 (p = 0.61). This did not appear to be due to the additive model being a poor fit for the effect of APOE, because when we utilized a two degree of freedom model for the ε3/ε4 effect, rs2075650 retained significance (p = 0.031) whereas neither the recessive nor dominance term for rs429358 was significant (p = 0.78 and p = 0.72, respectively). When rs2075650 and rs7412 were analyzed conditional on one another we found that both showed significant association with CAAD (p = 0.0047 for rs2075650 and p = 0.012 for rs7412). With a two degree of freedom model, rs2075650 retained significance (p = 0.0046) while the dominance term (p = 0.021) but not the recessive term (p = 0.40) was significant for rs7412. When all three SNPs were analyzed simultaneously including an interaction term for ε2/ε3 with ε3/ε4, rs2075650 was suggestively significant (p = 0.055), neither rs429358 nor its interaction with rs7412 was significant (p = 0.63 and p = 1.00, respectively), and rs7412 was significant (p = 0.036).

## Discussion

Our data confirm the effects of recently identified dyslipidemia SNPs, and we estimated that, after accounting for other covariates, genetic risk scores explain 3.9% and 3.3% of the variance in HDL and LDL, respectively. Although slightly different sets of SNPs and weighting factors were used, these values are in reasonable agreement with those of 4.8% and 3.4% as reported by Aulchenko et al[11]. Our estimates may be biased lower because of the extensive use of lipid lowering therapy in CLEAR participants with various pharmacological agents, dosing, and medication compliance which we have not attempted to fully model. In addition, while Aulchenko et al. sought to reduce upward bias by estimating weights for each SNP in independent cohorts from that in which the risk score was applied, the fact that the set of SNPs used in the risk score was apparently identified using all available cohorts suggests that a slight upward bias would still remain in their estimates. Based on the estimated effect sizes of this panel of SNPs on HDL and LDL, and the estimated effect size of dyslipidemia on CAAD, our data cannot exclude a model in which these SNPs as a group increase risk of CAAD mostly or entirely through their effects on lipids. However, our failure to identify a significant association between an overall risk score based on these SNPs and CAAD suggests that the majority of these SNPs are unlikely to contribute strongly to CAAD beyond their role in promoting dyslipidemia.

This result highlights a potential cause for caution in using a genetic risk score derived from an intermediate phenotype to predict disease. If genetic variants contributing to such a score act only through the intermediate clinical variable, then use of the clinical variable is likely to be superior. On the other hand, if genetic variants contribute to disease risk in ways not captured by the clinical variable, for example because they provide an index of lifetime exposure or because of pleiotropy with effects on the disease through multiple pathways, then a risk score may have significant clinical utility. However, when a score for an intermediate phenotype is developed using tens or hundreds of SNPs, it is likely that only a subset of the contributing SNPs will have these favorable predictive properties. Within the context of our study, we found no significant predictive power of the overall dyslipidemia risk score beyond what would be predicted by the role of dyslipidemia in CAAD alone, yet we identify two SNPs, rs646776 and rs2075650, which appear to mediate risk for CAAD beyond their effects on HDL and LDL. Based on its association with apo B, our analyses indicate that rs646776 affects LDL particle number in addition to LDL level, which may account for the additional explanatory power of this SNP. In contrast, rs2075650 appears to affect LDL buoyancy, with the minor allele contributing to the smaller, denser LDL particles that make up the more atherogenic LDL pattern B phenotype.

Based on our analyses of the 1p13.3 region containing rs646776, variation associated with CAAD is most likely located near CELSR2, a non-classical cadherin that does not interact with catenins, or PSRC1, a p53-regulated growth receptor. Although SORT1, a multi-ligand receptor present in the Golgi and on the cell surface, represents a good candidate gene because it binds and mediates degradation of lipoprotein lipase [26], increases its localization to the plasma membrane of adipocytes in response to insulin, and forms GLUT4 storage vesicles which enhance insulin sensitivity [27], our association signal was weaker in this gene. Given the strong linkage disequilibrium according to HapMap data, it is unlikely that there exists common variation in SORT1 that was not captured by our study. Although the 1p13.3 region appears to show robust association with coronary artery disease [28-30], a recent study that included 33,282 participants with a total of 503 strokes at baseline and 571 incident strokes did not identify a significant association between stroke and either rs599839 or rs4970834 in this region[31]. However, stroke is the sequela of a diverse set of underlying pathophysiologic causes which do not appear to have been distinguished in this study[32], so it is unclear whether this sample size would have sufficient power to detect an affect of the 1p13.3 region on the subset of strokes due to CAAD.

In the 19p13.2 region APOE is a stronger candidate gene than TOMM40, a channel forming subunit that is essential for protein import into the mitochondria [33]. The ε2/ε3/ε4 polymorphism is reported to be a equivocal risk factor for carotid atherosclerosis[10] although a more recent meta-analysis does support a modest association between these SNPs and carotid intima media thickness [34]. Consistent with this, our data show nominally significant protective effects of the ε2 allele and deleterious effects of the ε4 allele. However, conditioning on these SNPs did not account for the CAAD association signal in the region, and moreover the ε4 allele failed to demonstrate significant association with CAAD when rs2075650 was jointly considered. This result argues against a singular causal role for the APOE ε system in producing the CAAD association signal in this region, because one would expect the causal polymorphism to achieve greater statistical significance than, and in fact eliminate signal from, surrounding neutral variation. Instead, our data are most consistent with a causal polymorphism or collection of polymorphisms in linkage disequilibrium with both rs2075650 and the APOE ε system.

In summary, our data replicate the majority of associations reported between SNPs and HDL and LDL. Unbiased or slightly negatively biased estimates of the proportion of variance in HDL and LDL levels explained by these SNPs are 3.9% and 3.3% respectively, consistent with previous estimates[11]. The combined set of SNPs currently available does not improve CAAD risk prediction beyond what would be expected from their effects on HDL and LDL levels, but the specific SNPs rs646776 and rs2075650 are associated with CAAD risk, possibly due to their effects on LDL particle number and buoyancy, respectively.

## Methods

### Clear study participants

The Carotid Lesion Epidemiology And Risk (CLEAR) Study is a Seattle-based study involving the University of Washington (UW), Virginia Mason Medical Center (VM) and the Veterans Affairs Puget Sound Health Care System (VAPSHCS), focused on identifying predictors of CAAD, CAAD progression, and atherosclerotic plaque instability approved by the UW, VM, and VAPSHCS IRBs. All participants gave written informed consent. Participant characteristics are shown in Table 1. Only Caucasian males were analyzed due to under-representation of women and minorities in the cohort. Self reported ancestry was confirmed by STRUCTURE [35]. Individuals with total serum cholesterol >400 mg/dL or coagulopathy were excluded. Controls include 479 individuals with ≤15% carotid stenosis bilaterally as measured by duplex ultrasound. Individuals with vascular disease at other sites were excluded from the set of controls. Cases include 353 individuals status post carotid endarterectomy for symptomatic disease or asymptomatic individuals with ≥80% internal carotid stenosis either unilaterally or bilaterally. Individuals with intermediate stenosis have 50% to 79% luminal narrowing either unilaterally or bilaterally. Cases and controls were matched on age distribution, with censoring occurring at the time of diagnosis of vascular disease for cases or at the time of the last blood draw for controls. Hypertension was defined by treatment with antihypertensive medications. Diabetes was defined as a hemoglobin A1C≥6.5 or use of oral hypoglycemics or insulin.

### Lipid phenotypes

Standard methods were used to determine total cholesterol, triglycerides, and HDL in fasting whole plasma using an Abbott Spectrum analyzer. LDL was calculated unless triglycerides were ≥400 mg/dL, in which case it was measured directly. HDL fractions 2 and 3 were determined by precipitating HDL_2 _from total HDL, measuring HDL_3 _in the supernatant, and subtracting this from total HDL to obtain HDL_2_. Apolipoprotein A-I, apolipoprotein B, and lipoprotein(a) were measured as described by Marcovina et al [36], Zambon et al [37], and Marcovina et al [38], respectively. LDL buoyancy was measured by the relative flotation rate Rf as described by Capell et al [22]. We utilized lipid measurements prior to initiation of lipid lowering therapy whenever possible. For 90 individuals with two to three repeated lipid measurements we used the mean of these measurements. Based on inspection of the raw phenotype and residuals distributions, we excluded 6 outlying individuals with HDL>100 mg/dL, 10 individuals with HDL_2_>25 mg/dL, 4 individuals with HDL_3_>80, and 4 individuals with apo A-I>225 mg/dL. We also excluded 4 outlying individuals with LDL>200 mg/dL and 4 individuals with LDL fraction apolipoprotein B>120 mg/dL. The positively skewed lipoprotein (a) distribution was log transformed.

### Genotyping and SNPs

Genotypes were measured using the Illumina HumanCVD Genotyping BeadChip using an Illumina BeadStation Laboratory System platform[39,40]. Duplicate genotyping for 34 individuals showed 99.7% consistency in calls. The APOE ε2/ε3/ε4 polymorphism was genotyped as previously described [41]. Additional SNPs in the chromosome 1p13.3 and 19p13.2 regions were genotyped using TaqMan Assays by Design on an Applied Biosystems 7900HT System [42]. Using unphased reference genotypes from release 27 of the HapMap project [43]. we performed imputation for untyped SNPs using BIMBAM [44,45]. Although inaccurate genotype imputation is expected to cause false negatives rather than false positives, to avoid spurious conclusions we sought to determine which HapMap SNPs could be accurately imputed relying on the SNPs genotyped in the CLEAR study. We selected a random set of 10 individuals from the HapMap CEU sample and set to missing those SNPs not genotyped in the CLEAR study. We then imputed these missing SNPs using the remaining SNPs that had been genotyped in the CLEAR study. For each imputed SNP we computed the correlation between the imputed mean genotypes and the true genotypes for those HapMap individuals in whom genotypes had been masked. We repeated this procedure 20 times, selecting a different set of 10 individuals for genotype masking each time, and we report the imputation accuracy as the mean correlation over these 20 iterations. Only SNPs with >90% imputation accuracy were included in downstream analyses.

### Statistical analyses

All analyses were performed in R[46]. Unless otherwise specified, tests for genetic association were performed assuming an additive model with the homozygous genotypes coded as 0 or 2 and the heterozygous genotype coded as 1. Analyses of lipid phenotypes were performed in cases, individuals with intermediate stenosis, and controls using linear regression with censored age, body mass index (BMI), hypertension, diabetes, and use of lipid lowering therapy as covariates. Current cigarette usage was included as a covariate for analyses of HDL. Unless otherwise specified analyses of CAAD were performed using logistic regression with case status (≥80% stenosis) coded as 1 and control status (≤15% stenosis) coded as 0 and censored age, current cigarette usage, pack-years smoked, BMI, hypertension, and diabetes as covariates. To account for multiple testing we estimated false discovery rates (FDR) using the Benjamini-Hochberg procedure[47].

### Analysis of CAAD risk in the setting of lipid lowering therapy

Consistent with guidelines[18], cases with CAAD in the CLEAR study are generally treated with lipid lowering therapy to a target of LDL<100 mg/dL, whereas controls, who are without CAAD, coronary artery disease, or risk equivalents, are managed with a target LDL of <130 mg/dL or <160 mg/dL. Thus, including HDL, LDL, or lipid lowering therapy as covariates is problematic because it leads to a model in which the dependent variable, CAAD case control status, is causal for these independent variables. However, in order to study the effects of SNPs on CAAD in the context of lipid risk factors, we attempted to estimate "pre-therapy" HDL and LDL values for those individuals on lipid lowering therapy. We based on these values on the lipid altering effects of statins, the drug class for 92% of all lipid draws when therapy was in use. For HDL, we estimated that the post-therapy values were 2.1% to 9.6% higher than pre-therapy[48], and for LDL 30% to 63% lower than pre-therapy[48,49]. We also estimated the percent change in HDL (13% increase) and LDL (28% decrease) from 41 individuals in the CLEAR study who had measurements both prior to and following initiation of lipid lowering therapy.

### Power to detect association between genetic risk scores and CAAD

We performed simulations to determine our power to detect association between the genetic risk score and CAAD, given the percent variation in HDL and LDL levels explained by the genetic risk score and given the effect size of dyslipidemia as a risk factor for CAAD. Using the estimated "pre-therapy" HDL and LDL values described above, we first permuted the observed genotypes so that an additive score for the 16 HDL SNPs explained on average 3.9% of the variance in HDL levels and so that an additive score for the 18 LDL SNPs explained on average 3.3% of the variance in LDL levels. We then combined the HDL and LDL scores to form the overall genetic risk score. Next we simulated CAAD status by sampling binomial random variables with underlying probabilities given by the fitted effects of a logistic regression model that included "pre-therapy" HDL and LDL levels as well as all other covariates. For the three different "pre-therapy" HDL and LDL estimates described above, the odds ratios for association with CAAD in this model ranged from 0.95 to 0.96 per mg/dL change in HDL and 1.010 to 1.011 per mg/dL change in LDL. Finally, we tested for association between the simulated genetic risk score and simulated CAAD status in the setting of the usual covariates without HDL or LDL levels in the model. To estimate the expected odds ratio for the genetic risk score and the power, we performed 1000 such simulations for each of the three different estimates of "pre-therapy" HDL and LDL levels.

## Competing interests

The authors declare that they have no competing interests.

## Authors' contributions

JR and GPJ designed the study and wrote the manuscript. JR performed all statistical analyses with guidance from RR, PJH, and GPJ. JER performed the experimental work. JKM and TSH recruited and phenotyped study participants. All authors contributed to the interpretation of the data and approved the final version of this manuscript.

## References

[B1] Goldstein LB Adams R Alberts MJ Appel LJ Brass LM Bushnell CD Culebras A Degraba TJ Gorelick PB Guyton JR Hart RG Howard G Kelly-Hayes M Nixon JV Sacco RL American Heart Association/American Stroke Association Stroke Council; Atherosclerotic Peripheral Vascular Disease Interdisciplinary Working Group; Cardiovascular Nursing Council; Clinical Cardiology Council; Nutrition, Physical Activity, and Metabolism Council; Quality of Care and Outcomes Research Interdisciplinary Working Group; American Academy of Neurology Primary prevention of ischemic stroke: a guideline from the American Heart Association/American Stroke Association Stroke Council: cosponsored by the Atherosclerotic Peripheral Vascular Disease Interdisciplinary Working Group; Cardiovascular Nursing Council; Clinical Cardiology Council; Nutrition, Physical Activity, and Metabolism Council; and the Quality of Care and Outcomes Research Interdisciplinary Working Group: the American Academy of Neurology affirms the value of this guideline Stroke 2006 37 1583 1633 16675728 10.1161/01.STR.0000223048.70103.F1

[B2] Goldstein LB Adams R Becker K Furberg CD Gorelick PB Hademenos G Hill M Howard G Howard VJ Jacobs B Levine SR Mosca L Sacco RL Sherman DG Wolf PA del Zoppo GJ Primary prevention of ischemic stroke: A statement for healthcare professionals from the Stroke Council of the American Heart Association Stroke 2001 32 280 299 11136952 10.1161/01.str.32.1.280

[B3] Derdeyn CP Powers WJ Cost-effectiveness of screening for asymptomatic carotid atherosclerotic disease Stroke 1996 27 1944 1950 8898796 10.1161/01.str.27.11.1944

[B4] Whitty CJ Sudlow CL Warlow CP Investigating individual subjects and screening populations for asymptomatic carotid stenosis can be harmful J Neurol Neurosurg Psychiatry 1998 64 619 623 10.1136/jnnp.64.5.619 9598677 PMC2170073

[B5] North KE MacCluer JW Devereux RB Howard BV Welty TK Best LG Lee ET Fabsitz RR Roman MJ Heritability of carotid artery structure and function: the Strong Heart Family Study Arterioscler Thromb Vasc Biol 2002 22 1698 1703 10.1161/01.ATV.0000032656.91352.5E 12377752

[B6] Zannad F Visvikis S Gueguen R Sass C Chapet O Herbeth B Siest G Genetics strongly determines the wall thickness of the left and right carotid arteries Hum Genet 1998 103 183 188 10.1007/s004390050804 9760203

[B7] Jartti L Ronnemaa T Kaprio J Jarvisalo MJ Toikka JO Marniemi J Hammar N Alfredsson L Saraste M Hartiala J Koskenvuo M Raitakari OT Population-based twin study of the effects of migration from Finland to Sweden on endothelial function and intima-media thickness Arterioscler Thromb Vasc Biol 2002 22 832 837 10.1161/01.ATV.0000013313.70875.A7 12006398

[B8] Lange LA Bowden DW Langefeld CD Wagenknecht LE Carr JJ Rich SS Riley WA Freedman BI Heritability of carotid artery intima-medial thickness in type 2 diabetes Stroke 2002 33 1876 1881 10.1161/01.STR.0000019909.71547.AA 12105369

[B9] Fox CS Polak JF Chazaro I Cupples A Wolf PA D'Agostino RA O'Donnell CJ Genetic and environmental contributions to atherosclerosis phenotypes in men and women: heritability of carotid intima-media thickness in the Framingham Heart Study Stroke 2003 34 397 401 10.1161/01.STR.0000048214.56981.6F 12574549

[B10] Manolio TA Boerwinkle E O'Donnell CJ Wilson AF Genetics of ultrasonographic carotid atherosclerosis Arterioscler Thromb Vasc Biol 2004 24 1567 1577 10.1161/01.ATV.0000138789.11433.c1 15256397

[B11] Aulchenko YS Ripatti S Lindqvist I Boomsma D Heid IM Pramstaller PP Penninx BW Janssens AC Wilson JF Spector T Loci influencing lipid levels and coronary heart disease risk in 16 European population cohorts Nat Genet 2009 41 47 55 10.1038/ng.269 19060911 PMC2687074

[B12] Kathiresan S Melander O Anevski D Guiducci C Burtt NP Roos C Hirschhorn JN Berglund G Hedblad B Groop L Altshuler DM Newton-Cheh C Orho-Melander M Polymorphisms associated with cholesterol and risk of cardiovascular events N Engl J Med 2008 358 1240 1249 10.1056/NEJMoa0706728 18354102

[B13] Kathiresan S Melander O Guiducci C Surti A Burtt NP Rieder MJ Cooper GM Roos C Voight BF Havulinna AS Wahlstrand B Hedner T Corella D Tai ES Ordovas JM Berglund G Vartiainen E Jousilahti P Hedblad B Taskinen MR Newton-Cheh C Salomaa V Peltonen L Groop L Altshuler DM Orho-Melander M Six new loci associated with blood low-density lipoprotein cholesterol, high-density lipoprotein cholesterol or triglycerides in humans Nat Genet 2008 40 189 197 10.1038/ng.75 18193044 PMC2682493

[B14] Kathiresan S Willer CJ Peloso GM Demissie S Musunuru K Schadt EE Kaplan L Bennett D Li Y Tanaka T Common variants at 30 loci contribute to polygenic dyslipidemia Nat Genet 2009 41 56 65 10.1038/ng.291 19060906 PMC2881676

[B15] Willer CJ Sanna S Jackson AU Scuteri A Bonnycastle LL Clarke R Heath SC Timpson NJ Najjar SS Stringham HM Newly identified loci that influence lipid concentrations and risk of coronary artery disease Nat Genet 2008 40 161 169 10.1038/ng.76 18193043 PMC5206900

[B16] Samani NJ Deloukas P Erdmann J Hengstenberg C Kuulasmaa K McGinnis R Schunkert H Soranzo N Thompson J Tiret L Ziegler A Large scale association analysis of novel genetic loci for coronary artery disease Arterioscler Thromb Vasc Biol 2009 29 774 780 10.1161/ATVBAHA.108.181388 19164808 PMC3315048

[B17] Sharrett AR Sorlie PD Chambless LE Folsom AR Hutchinson RG Heiss G Szklo M Relative importance of various risk factors for asymptomatic carotid atherosclerosis versus coronary heart disease incidence: the Atherosclerosis Risk in Communities Study Am J Epidemiol 1999 149 843 852 10221321 10.1093/oxfordjournals.aje.a009900

[B18] National Cholesterol Education Program (NCEP) Expert Panel on Detection Evaluation and Treatment of High Blood Cholesterol in Adults (Adult Treatment Panel III) Third Report of the National Cholesterol Education Program (NCEP) Expert Panel on Detection, Evaluation, and Treatment of High Blood Cholesterol in Adults (Adult Treatment Panel III) final report Circulation 2002 106 3143 3421 12485966

[B19] Huang Y von Eckardstein A Wu S Assmann G Cholesterol efflux, cholesterol esterification, and cholesteryl ester transfer by LpA-I and LpA-I/A-II in native plasma Arterioscler Thromb Vasc Biol 1995 15 1412 1418 7670956 10.1161/01.atv.15.9.1412

[B20] Barter PJ Ballantyne CM Carmena R Castro Cabezas M Chapman MJ Couture P de Graaf J Durrington PN Faergeman O Frohlich J Furberg CD Gagne C Haffner SM Humphries SE Jungner I Krauss RM Kwiterovich P Marcovina S Packard CJ Pearson TA Reddy KS Rosenson R Sarrafzadegan N Sniderman AD Stalenhoef AF Stein E Talmud PJ Tonkin AM Walldius G Williams KM Apo B versus cholesterol in estimating cardiovascular risk and in guiding therapy: report of the thirty-person/ten-country panel J Intern Med 2006 259 247 258 10.1111/j.1365-2796.2006.01616.x 16476102

[B21] Benn M Nordestgaard BG Jensen GB Tybjaerg-Hansen A Improving prediction of ischemic cardiovascular disease in the general population using apolipoprotein B: the Copenhagen City Heart Study Arterioscler Thromb Vasc Biol 2007 27 661 670 10.1161/01.ATV.0000255580.73689.8e 17170368

[B22] Capell WH Zambon A Austin MA Brunzell JD Hokanson JE Compositional differences of LDL particles in normal subjects with LDL subclass phenotype A and LDL subclass phenotype B Arterioscler Thromb Vasc Biol 1996 16 1040 1046 8696944 10.1161/01.atv.16.8.1040

[B23] St-Pierre AC Ruel IL Cantin B Dagenais GR Bernard PM Despres JP Lamarche B Comparison of various electrophoretic characteristics of LDL particles and their relationship to the risk of ischemic heart disease Circulation 2001 104 2295 2299 10.1161/hc4401.098490 11696468

[B24] Danesh J Collins R Peto R Lipoprotein(a) and coronary heart disease. Meta-analysis of prospective studies Circulation 2000 102 1082 1085 10973834 10.1161/01.cir.102.10.1082

[B25] Smolders B Lemmens R Thijs V Lipoprotein (a) and stroke: a meta-analysis of observational studies Stroke 2007 38 1959 1966 10.1161/STROKEAHA.106.480657 17478739

[B26] Nielsen MS Jacobsen C Olivecrona G Gliemann J Petersen CM Sortilin/neurotensin receptor-3 binds and mediates degradation of lipoprotein lipase J Biol Chem 1999 274 8832 8836 10.1074/jbc.274.13.8832 10085125

[B27] Shi J Kandror KV Sortilin is essential and sufficient for the formation of Glut4 storage vesicles in 3T3-L1 adipocytes Dev Cell 2005 9 99 108 10.1016/j.devcel.2005.04.004 15992544

[B28] Muendlein A Geller-Rhomberg S Saely CH Winder T Sonderegger G Rein P Beer S Vonbank A Drexel H Significant impact of chromosomal locus 1p13.3 on serum LDL cholesterol and on angiographically characterized coronary atherosclerosis Atherosclerosis 2009 206 2 494 9 10.1016/j.atherosclerosis.2009.02.040 19380133

[B29] Samani NJ Braund PS Erdmann J Gotz A Tomaszewski M Linsel-Nitschke P Hajat C Mangino M Hengstenberg C Stark K Ziegler A Caulfield M Burton PR Schunkert H Tobin MD The novel genetic variant predisposing to coronary artery disease in the region of the PSRC1 and CELSR2 genes on chromosome 1 associates with serum cholesterol J Mol Med 2008 86 1233 1241 10.1007/s00109-008-0387-2 18649068

[B30] Samani NJ Erdmann J Hall AS Hengstenberg C Mangino M Mayer B Dixon RJ Meitinger T Braund P Wichmann HE Barrett JH König IR Stevens SE Szymczak S Tregouet DA Iles MM Pahlke F Pollard H Lieb W Cambien F Fischer M Ouwehand W Blankenberg S Balmforth AJ Baessler A Ball SG Strom TM Braenne I Gieger C Deloukas P Tobin MD Ziegler A Thompson JR Schunkert H WTCCC and the Cardiogenics Consortium Genomewide association analysis of coronary artery disease N Engl J Med 2007 357 443 453 10.1056/NEJMoa072366 17634449 PMC2719290

[B31] Karvanen J Silander K Kee F Tiret L Salomaa V Kuulasmaa K Wiklund PG Virtamo J Saarela O Perret C Perola M Peltonen L Cambien F Erdmann J Samani NJ Schunkert H Evans A MORGAM Project The impact of newly identified loci on coronary heart disease, stroke and total mortality in the MORGAM prospective cohorts Genet Epidemiol 2009 33 237 246 10.1002/gepi.20374 18979498 PMC2696097

[B32] Asplund K Karvanen J Giampaoli S Jousilahti P Niemela M Broda G Cesana G Dallongeville J Ducimetriere P Evans A Ferrières J Haas B Jorgensen T Tamosiunas A Vanuzzo D Wiklund PG Yarnell J Kuulasmaa K Kulathinal S MORGAM Project Relative risks for stroke by age, sex, and population based on follow-up of 18 European populations in the MORGAM Project Stroke 2009 40 2319 2326 10.1161/STROKEAHA.109.547869 19520994

[B33] Humphries AD Streimann IC Stojanovski D Johnston AJ Yano M Hoogenraad NJ Ryan MT Dissection of the mitochondrial import and assembly pathway for human Tom40 J Biol Chem 2005 280 11535 11543 10.1074/jbc.M413816200 15644312

[B34] Paternoster L Martinez Gonzalez NA Lewis S Sudlow C Association between apolipoprotein E genotype and carotid intima-media thickness may suggest a specific effect on large artery atherothrombotic stroke Stroke 2008 39 48 54 10.1161/STROKEAHA.107.488866 18063831 PMC2577179

[B35] Pritchard JK Stephens M Donnelly P Inference of population structure using multilocus genotype data Genetics 2000 155 945 959 10835412 10.1093/genetics/155.2.945 PMC1461096

[B36] Marcovina SM Albers JJ Henderson LO Hannon WH International Federation of Clinical Chemistry standardization project for measurements of apolipoproteins A-I and B. III. Comparability of apolipoprotein A-I values by use of international reference material Clin Chem 1993 39 773 781 8485867

[B37] Zambon A Austin MA Brown BG Hokanson JE Brunzell JD Effect of hepatic lipase on LDL in normal men and those with coronary artery disease Arterioscler Thromb 1993 13 147 153 8427851 10.1161/01.atv.13.2.147

[B38] Marcovina SM Albers JJ Gabel B Koschinsky ML Gaur VP Effect of the number of apolipoprotein(a) kringle 4 domains on immunochemical measurements of lipoprotein(a) Clin Chem 1995 41 246 255 7533064

[B39] Fan JB Oliphant A Shen R Kermani BG Garcia F Gunderson KL Hansen M Steemers F Butler SL Deloukas P Galver L Hunt S McBride C Bibikova M Rubano T Chen J Wickham E Doucet D Chang W Campbell D Zhang B Kruglyak S Bentley D Haas J Rigault P Zhou L Stuelpnagel J Chee MS Highly parallel SNP genotyping Cold Spring Harb Symp Quant Biol 2003 68 69 78 10.1101/sqb.2003.68.69 15338605

[B40] Keating BJ Tischfield S Murray SS Bhangale T Price TS Glessner JT Galver L Barrett JC Grant SF Farlow DN Concept, design and implementation of a cardiovascular gene-centric 50 k SNP array for large-scale genomic association studies PLoS One 2008 3 e3583 10.1371/journal.pone.0003583 18974833 PMC2571995

[B41] Yu CE Devlin B Galloway N Loomis E Schellenberg GD ADLAPH: A molecular haplotyping method based on allele-discriminating long-range PCR Genomics 2004 84 600 612 10.1016/j.ygeno.2004.06.003 15498468

[B42] De la Vega FM Lazaruk KD Rhodes MD Wenz MH Assessment of two flexible and compatible SNP genotyping platforms: TaqMan SNP Genotyping Assays and the SNPlex Genotyping System Mutat Res 2005 573 111 135 15829242 10.1016/j.mrfmmm.2005.01.008

[B43] The International HapMap Consortium The International HapMap Project Nature 2003 426 789 796 10.1038/nature02168 14685227

[B44] Guan Y Stephens M Practical issues in imputation-based association mapping PLoS Genet 2008 4 e1000279 10.1371/journal.pgen.1000279 19057666 PMC2585794

[B45] Scheet P Stephens M A fast and flexible statistical model for large-scale population genotype data: applications to inferring missing genotypes and haplotypic phase Am J Hum Genet 2006 78 629 644 10.1086/502802 16532393 PMC1424677

[B46] R: A language and environment for statistical computing http://www.R-project.org

[B47] Benjamini Y Hochberg Y Controlling the false discovery rate: a practical and powerful approach to multiple testing J Roy Statist Soc Ser B 1995 57 289 300

[B48] Jones PH Davidson MH Stein EA Bays HE McKenney JM Miller E Cain VA Blasetto JW Comparison of the efficacy and safety of rosuvastatin versus atorvastatin, simvastatin, and pravastatin across doses (STELLAR* Trial) Am J Cardiol 2003 92 152 160 10.1016/S0002-9149(03)00530-7 12860216

[B49] Jones P Kafonek S Laurora I Hunninghake D Comparative dose efficacy study of atorvastatin versus simvastatin, pravastatin, lovastatin, and fluvastatin in patients with hypercholesterolemia (the CURVES study) Am J Cardiol 1998 81 582 587 10.1016/S0002-9149(97)00965-X 9514454

